# Ethyl 2-(3-amino-4-hy­droxy­phen­yl)acetate

**DOI:** 10.1107/S1600536810044399

**Published:** 2010-11-06

**Authors:** Fang Zhang, Wen-De Liang, Gong-Xing Li, Wang Jiang, Zhu-Ping Xiao

**Affiliations:** a College of Chemistry & Chemical Engineering, Jishou University, Jishou 416000, People’s Republic of China

## Abstract

The asymmetric unit of the title compound, C_10_H_13_NO_3_, contains two crystallographically independent mol­ecules with different conformations of the eth­oxy­carbonyl groups; the terminal C—C—O—C torsion angles in the two mol­ecules are 83.6 (6) and −171.1 (3)°, resulting in twisted and straight chain conformations, respectively. The crystal structure is stabilized by inter­molecular N—H⋯O, O—H⋯N and C—H⋯O hydrogen bonds. Intra­molecular hydrogen bonds occur between the amino N and phenolic O atoms.

## Related literature

For general background to the use of phenyl­acetate derivatives as inter­mediates for the rational design of new chemotherapeutic agents, see: Xiao, Fang *et al.* (2008 ▶); Xiao, Lv *et al.* (2008 ▶). For the preparation of the title compound, see: Xiao *et al.* 2010 ▶. For bond-length data, see: Allen *et al.* (1987 ▶).
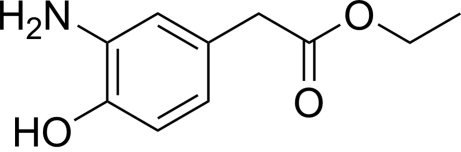


## Experimental

### 

#### Crystal data

C_10_H_13_NO_3_
*M* *_r_* = 195.21
Triclinic, 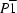

*a* = 8.5940 (17) Å
*b* = 10.142 (2) Å
*c* = 12.043 (2) Å
α = 98.23 (3)°
β = 104.96 (3)°
γ = 90.41 (3)°
*V* = 1002.6 (3) Å^3^
*Z* = 4
Mo *K*α radiation
μ = 0.10 mm^−1^
*T* = 293 K
0.30 × 0.10 × 0.10 mm

#### Data collection

Bruker SMART APEX CCD diffractometer
Absorption correction: multi-scan (*SADABS*; Sheldrick, 1996 ▶) *T*_min_ = 0.972, *T*_max_ = 0.991
3857 measured reflections
3598 independent reflections
2121 reflections with *I* > 2σ(*I*)
*R*_int_ = 0.030

#### Refinement

*R*[*F* ^2^ > 2σ(*F* ^2^)] = 0.079
*wR*(*F* ^2^) = 0.213
*S* = 1.09
3598 reflections
247 parameters
H-atom parameters constrained
Δρ_max_ = 0.32 e Å^−3^
Δρ_min_ = −0.32 e Å^−3^

### 

Data collection: *SMART* (Bruker, 2007 ▶); cell refinement: *SAINT* (Bruker, 2007 ▶); data reduction: *SAINT*; program(s) used to solve structure: *SHELXS97* (Sheldrick, 2008 ▶); program(s) used to refine structure: *SHELXL97* (Sheldrick, 2008 ▶); molecular graphics: *SHELXTL* (Sheldrick, 2008 ▶); software used to prepare material for publication: *SHELXL97*.

## Supplementary Material

Crystal structure: contains datablocks global, I. DOI: 10.1107/S1600536810044399/pv2339sup1.cif

Structure factors: contains datablocks I. DOI: 10.1107/S1600536810044399/pv2339Isup2.hkl

Additional supplementary materials:  crystallographic information; 3D view; checkCIF report

## Figures and Tables

**Table 1:** Hydrogen-bond geometry (Å, °)

*D*—H⋯*A*	*D*—H	H⋯*A*	*D*⋯*A*	*D*—H⋯*A*
N1—H1*B*⋯O2^i^	0.86	2.37	3.097 (5)	143
N2—H2*D*⋯O5^ii^	0.86	2.42	3.222 (5)	155
O3—H3*A*⋯N2^iii^	0.82	2.23	2.978 (5)	152
O6—H6*B*⋯N1^iv^	0.82	2.31	3.015 (5)	145
C10—H10*A*⋯O6^ii^	0.93	2.49	3.414 (5)	174
N1—H1*B*⋯O3	0.86	2.12	2.517 (5)	108
N2—H2*D*⋯O6	0.86	2.33	2.641 (4)	102

Symmetry codes: (i) 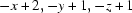; (ii) 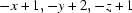; (iii) 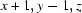; (iv) 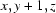.

**Fig. 1.**
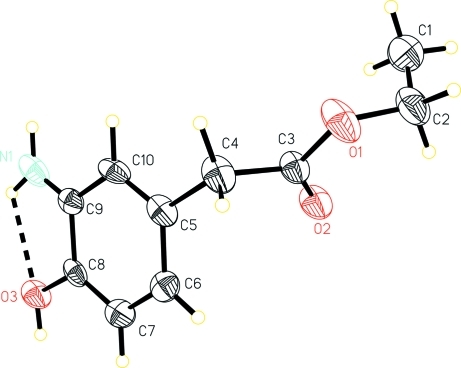
A view of the molecule (1) of the title compound, showing the atom-labelling scheme. Displacement ellipsoids are drawn at the 30% probability level.

**Fig. 2.**
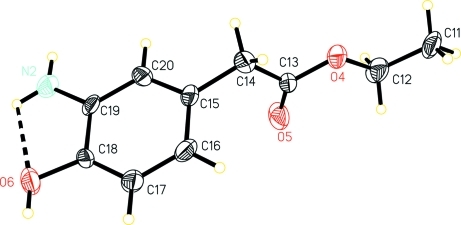
A view of the molecule (2) of the title compound, showing the atom-labelling scheme. Displacement ellipsoids are drawn at the 30% probability level.

**Fig. 3.**
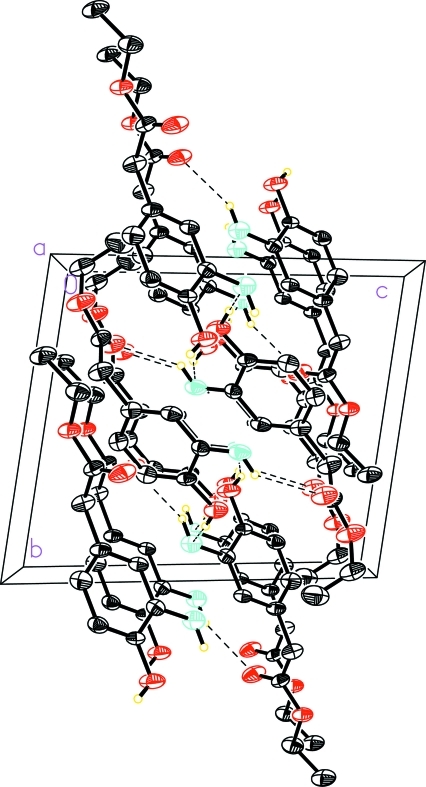
A unit cell packing of the title compound showing intermolecular N—H···O and O—H···N hydrogen bonds by dashed lines.

## supplementary crystallographic information

### Comment

Derivatives of phenylacetate are key intermates for the rational design of new chemotherapeutic agents such as antibacterials (Xiao, Fang, *et al.* 2008) and anticancers (Xiao, Lv, *et al.* 2008). As a part of our research on pharmoceutically active 4-hydroxy-3-phenylfuran-2(5*H*)-ones, we have synthesized the title compound and herein we report its crystal structure.

The crystal structure contains two crystallographically independent molecules in an asymmetric unit of the title compound with different conformations of the ethoxy carbonyl groups. The terminal torsion angles C1—C2—O1—C3 in molecule (1) and C11—C12—O4—C13 in molecule (2) are 83.6 (6) and -171.1 (3)° resulting in twisted and straight chian conformations, respectively (Figures 1 and 2). In both molecules of the title compound, the bond lengths and angles are within normal ranges (Allen *et al.*, 1987). The molecules assemble into an infinite two-dimensional ribbon through intermolecular N—H···O, O—H···N and C—H···O hydrogen bonds (Table 1 and Fig. 3). In each molecule there is an intramolecular N—H···O hydrogen bond resulting in a five-membered ring (Table 2 and Fig. 1 and 2).

### Experimental

The title compound was prepared according to the reported procedures (Xiao *et al.*, 2010). Reduced iron powder was added to a solution of ammonium chloride (0.8 g, 15 mmole) in water (10 ml) under nitrogen atmosphere. To this stirring mixture, a solution of ethyl 2-(4-hydroxy-3-nitrophenyl)acetate (1.13 g, 5 mmole) in acetone (25 ml) was added dropwise. It was refluxed in an oil bath for 4 h. When the reaction was complete, the resulting mixture was extracted with ethylacetate, and the combined organic layers were basified by adding a saturated solution of NaHCO_3_. The solvent was removed under reduced pressure and furnished the title compound, which was crystallized from ethylacetate-petroleum ether (1:2) to give colorless blocks suitable for single-crystal structure determination.

### Refinement

The H atoms were placed in geometrically idealized positions and constrained to ride on their parent atoms with N—H = 0.86 and O—H = 0.82 Å and C—H = 0.93, 0.97 and 0.96 Å for aryl, methyl and methylene groups, reaspectively. *U*_iso_(H) values were set at 1.5 × *U*_eq_(O and methyl C) and 1.2 × the *U*_eq_ of the rest of the parent atoms.

### Crystal data

**Table:**

C_10_H_13_NO_3_	*Z* = 4
*M**_r_* = 195.21	*F*(000) = 416
Triclinic, *P*1	*D*_x_ = 1.293 Mg m^−^^3^
Hall symbol: -P 1	Mo *K*α radiation, λ = 0.71073 Å
*a* = 8.5940 (17) Å	Cell parameters from 2097 reflections
*b* = 10.142 (2) Å	θ = 2.4–24.9°
*c* = 12.043 (2) Å	µ = 0.10 mm^−^^1^
α = 98.23 (3)°	*T* = 293 K
β = 104.96 (3)°	Block, colorless
γ = 90.41 (3)°	0.30 × 0.10 × 0.10 mm
*V* = 1002.6 (3) Å^3^	

### Data collection

**Table:**

Bruker SMART APEX CCD diffractometer	3598 independent reflections
Radiation source: fine-focus sealed tube	2121 reflections with *I* > 2σ(*I*)
graphite	*R*_int_ = 0.030
φ and ω scans	θ_max_ = 25.2°, θ_min_ = 1.8°
Absorption correction: multi-scan (*SADABS*; Sheldrick, 1996)	*h* = −10→9
*T*_min_ = 0.972, *T*_max_ = 0.991	*k* = −12→12
3857 measured reflections	*l* = 0→14

### Refinement

**Table:**

Refinement on *F*^2^	Primary atom site location: structure-invariant direct methods
Least-squares matrix: full	Secondary atom site location: difference Fourier map
*R*[*F*^2^ > 2σ(*F*^2^)] = 0.079	Hydrogen site location: inferred from neighbouring sites
*wR*(*F*^2^) = 0.213	H-atom parameters constrained
*S* = 1.09	*w* = 1/[σ^2^(*F*_o_^2^) + (0.0847*P*)^2^ + 0.7608*P*] where *P* = (*F*_o_^2^ + 2*F*_c_^2^)/3
3598 reflections	(Δ/σ)_max_ < 0.001
247 parameters	Δρ_max_ = 0.32 e Å^−^^3^
0 restraints	Δρ_min_ = −0.31 e Å^−^^3^

### Special details

**Table:**

Geometry. All e.s.d.'s (except the e.s.d. in the dihedral angle between two l.s. planes) are estimated using the full covariance matrix. The cell e.s.d.'s are taken into account individually in the estimation of e.s.d.'s in distances, angles and torsion angles; correlations between e.s.d.'s in cell parameters are only used when they are defined by crystal symmetry. An approximate (isotropic) treatment of cell e.s.d.'s is used for estimating e.s.d.'s involving l.s. planes.
Refinement. Refinement of *F*^2^ against ALL reflections. The weighted *R*-factor *wR* and goodness of fit *S* are based on *F*^2^, conventional *R*-factors *R* are based on *F*, with *F* set to zero for negative *F*^2^. The threshold expression of *F*^2^ > σ(*F*^2^) is used only for calculating *R*-factors(gt) *etc*. and is not relevant to the choice of reflections for refinement. *R*-factors based on *F*^2^ are statistically about twice as large as those based on *F*, and *R*- factors based on ALL data will be even larger.

### Fractional atomic coordinates and isotropic or equivalent isotropic displacement parameters (Å^2^)

**Table:**

	*x*	*y*	*z*	*U*_iso_*/*U*_eq_	
O1	1.0536 (4)	0.8579 (3)	0.9060 (3)	0.0860 (11)	
O2	1.2035 (3)	0.7342 (3)	0.8128 (3)	0.0690 (9)	
O3	0.9659 (3)	0.2198 (3)	0.4667 (3)	0.0611 (8)	
H3A	1.0353	0.1671	0.4878	0.092*	
N1	0.7667 (5)	0.3994 (3)	0.4283 (3)	0.063	
H1A	0.6993	0.4586	0.4059	0.075*	
H1B	0.7774	0.3314	0.3797	0.075*	
C1	1.1307 (7)	1.0449 (5)	0.8320 (6)	0.102 (2)	
H1C	1.2096	1.1172	0.8466	0.153*	
H1D	1.0260	1.0801	0.8263	0.153*	
H1E	1.1306	0.9893	0.7604	0.153*	
C2	1.1679 (6)	0.9708 (5)	0.9218 (5)	0.0779 (15)	
H2A	1.1697	1.0281	0.9939	0.093*	
H2B	1.2750	0.9377	0.9283	0.093*	
C3	1.0796 (5)	0.7484 (4)	0.8404 (4)	0.0513 (10)	
C4	0.9484 (5)	0.6448 (4)	0.8233 (4)	0.0655 (13)	
H4A	0.8454	0.6861	0.8036	0.079*	
H4B	0.9581	0.6112	0.8962	0.079*	
C5	0.9492 (5)	0.5276 (4)	0.7290 (4)	0.0500 (10)	
C6	1.0634 (5)	0.4308 (4)	0.7533 (4)	0.0514 (10)	
H6A	1.1342	0.4357	0.8267	0.062*	
C7	1.0690 (5)	0.3282 (4)	0.6664 (4)	0.0531 (10)	
H7A	1.1455	0.2644	0.6825	0.064*	
C8	0.9663 (4)	0.3164 (3)	0.5569 (3)	0.0430 (9)	
C9	0.8494 (4)	0.4121 (3)	0.5322 (3)	0.0424 (9)	
C10	0.8459 (5)	0.5164 (4)	0.6213 (4)	0.0498 (10)	
H10A	0.7697	0.5806	0.6061	0.060*	
O4	0.3631 (3)	0.5329 (2)	0.1330 (2)	0.0541 (7)	
O5	0.5351 (4)	0.6553 (3)	0.2847 (3)	0.0739 (10)	
O6	0.4558 (4)	1.2663 (3)	0.4366 (3)	0.0727 (10)	
H6B	0.5107	1.3115	0.4082	0.087*	
N2	0.2886 (4)	1.1022 (4)	0.5184 (3)	0.0628 (10)	
H2C	0.2324	1.0498	0.5447	0.075*	
H2D	0.3165	1.1816	0.5548	0.075*	
C11	0.4060 (6)	0.3167 (4)	0.0431 (4)	0.0734 (14)	
H11A	0.4722	0.2412	0.0527	0.110*	
H11B	0.4119	0.3505	−0.0263	0.110*	
H11C	0.2963	0.2901	0.0369	0.110*	
C12	0.4636 (5)	0.4217 (4)	0.1442 (4)	0.0642 (12)	
H12A	0.4625	0.3866	0.2148	0.077*	
H12B	0.5737	0.4501	0.1496	0.077*	
C13	0.4116 (5)	0.6431 (4)	0.2094 (3)	0.0465 (9)	
C14	0.2909 (5)	0.7474 (4)	0.1919 (4)	0.0570 (11)	
H14A	0.1973	0.7178	0.2141	0.068*	
H14B	0.2568	0.7527	0.1094	0.068*	
C15	0.3408 (4)	0.8849 (3)	0.2550 (3)	0.0454 (9)	
C16	0.4443 (5)	0.9678 (4)	0.2213 (4)	0.0559 (11)	
H16A	0.4852	0.9373	0.1584	0.067*	
C17	0.4866 (5)	1.0952 (4)	0.2809 (3)	0.0508 (10)	
H17A	0.5512	1.1512	0.2548	0.061*	
C18	0.4349 (4)	1.1409 (3)	0.3781 (3)	0.0374 (8)	
C19	0.3347 (4)	1.0583 (3)	0.4161 (3)	0.0403 (8)	
C20	0.2923 (4)	0.9312 (3)	0.3547 (3)	0.0443 (9)	
H20A	0.2290	0.8746	0.3812	0.053*	

### Atomic displacement parameters (Å^2^)

**Table:**

	*U*^11^	*U*^22^	*U*^33^	*U*^12^	*U*^13^	*U*^23^
O1	0.090 (2)	0.0554 (19)	0.127 (3)	−0.0022 (17)	0.072 (2)	−0.0197 (19)
O2	0.0548 (18)	0.0541 (18)	0.104 (2)	0.0050 (14)	0.0410 (17)	−0.0067 (16)
O3	0.0565 (17)	0.0441 (16)	0.092 (2)	0.0117 (13)	0.0403 (15)	0.0021 (15)
N1	0.082	0.039	0.083	−0.002	0.049	0.011
C1	0.092 (4)	0.067 (3)	0.132 (5)	−0.005 (3)	−0.001 (4)	0.023 (4)
C2	0.080 (3)	0.054 (3)	0.099 (4)	−0.001 (3)	0.036 (3)	−0.014 (3)
C3	0.051 (2)	0.045 (2)	0.063 (3)	0.0007 (18)	0.030 (2)	−0.0049 (19)
C4	0.057 (3)	0.056 (3)	0.093 (3)	0.000 (2)	0.048 (2)	−0.011 (2)
C5	0.050 (2)	0.042 (2)	0.072 (3)	0.0019 (18)	0.044 (2)	0.0027 (19)
C6	0.042 (2)	0.056 (3)	0.060 (2)	0.0005 (18)	0.0209 (18)	0.008 (2)
C7	0.042 (2)	0.051 (2)	0.077 (3)	0.0067 (17)	0.032 (2)	0.015 (2)
C8	0.045 (2)	0.0288 (18)	0.064 (3)	−0.0039 (15)	0.0325 (19)	0.0027 (17)
C9	0.043 (2)	0.039 (2)	0.054 (2)	−0.0008 (16)	0.0248 (17)	0.0128 (17)
C10	0.052 (2)	0.035 (2)	0.077 (3)	0.0142 (17)	0.042 (2)	0.0121 (19)
O4	0.0550 (16)	0.0347 (14)	0.0755 (19)	0.0100 (12)	0.0259 (14)	0.0012 (13)
O5	0.0589 (19)	0.0487 (17)	0.099 (2)	0.0180 (14)	0.0063 (18)	−0.0132 (16)
O6	0.103 (2)	0.0329 (15)	0.098 (2)	−0.0021 (15)	0.063 (2)	−0.0031 (15)
N2	0.072 (2)	0.054 (2)	0.080 (3)	0.0021 (17)	0.052 (2)	0.0063 (18)
C11	0.086 (3)	0.048 (3)	0.103 (4)	0.012 (2)	0.064 (3)	−0.007 (2)
C12	0.061 (3)	0.049 (2)	0.090 (3)	0.020 (2)	0.031 (2)	0.012 (2)
C13	0.049 (2)	0.036 (2)	0.058 (2)	−0.0049 (17)	0.0269 (19)	−0.0070 (17)
C14	0.046 (2)	0.044 (2)	0.077 (3)	−0.0004 (18)	0.016 (2)	−0.006 (2)
C15	0.042 (2)	0.0305 (19)	0.068 (3)	0.0096 (15)	0.0244 (18)	0.0029 (17)
C16	0.054 (2)	0.052 (2)	0.077 (3)	0.0091 (19)	0.042 (2)	0.010 (2)
C17	0.059 (2)	0.040 (2)	0.065 (3)	0.0037 (18)	0.034 (2)	0.0120 (19)
C18	0.0343 (18)	0.0245 (17)	0.053 (2)	0.0034 (13)	0.0115 (15)	0.0042 (15)
C19	0.0350 (18)	0.038 (2)	0.055 (2)	0.0165 (15)	0.0225 (16)	0.0106 (17)
C20	0.044 (2)	0.0311 (19)	0.060 (2)	−0.0016 (15)	0.0172 (18)	0.0090 (17)

### Geometric parameters (Å, °)

**Table:**

O1—C3	1.324 (5)	O4—C13	1.331 (4)
O1—C2	1.462 (6)	O4—C12	1.426 (5)
O2—C3	1.199 (4)	O5—C13	1.198 (5)
O3—C8	1.353 (4)	O6—C18	1.350 (4)
O3—H3A	0.8200	O6—H6B	0.8200
N1—C9	1.257 (5)	N2—C19	1.405 (5)
N1—H1A	0.8600	N2—H2C	0.8600
N1—H1B	0.8600	N2—H2D	0.8600
C1—C2	1.376 (7)	C11—C12	1.474 (6)
C1—H1C	0.9600	C11—H11A	0.9600
C1—H1D	0.9600	C11—H11B	0.9600
C1—H1E	0.9600	C11—H11C	0.9600
C2—H2A	0.9700	C12—H12A	0.9700
C2—H2B	0.9700	C12—H12B	0.9700
C3—C4	1.489 (5)	C13—C14	1.485 (6)
C4—C5	1.523 (5)	C14—C15	1.491 (5)
C4—H4A	0.9700	C14—H14A	0.9700
C4—H4B	0.9700	C14—H14B	0.9700
C5—C10	1.359 (6)	C15—C20	1.391 (5)
C5—C6	1.400 (6)	C15—C16	1.391 (5)
C6—C7	1.377 (5)	C16—C17	1.382 (5)
C6—H6A	0.9300	C16—H16A	0.9300
C7—C8	1.372 (6)	C17—C18	1.379 (5)
C7—H7A	0.9300	C17—H17A	0.9300
C8—C9	1.408 (5)	C18—C19	1.398 (5)
C9—C10	1.401 (5)	C19—C20	1.386 (5)
C10—H10A	0.9300	C20—H20A	0.9300
			
C3—O1—C2	116.0 (3)	C13—O4—C12	117.4 (3)
C8—O3—H3A	109.5	C18—O6—H6B	109.5
C9—N1—H1A	120.0	C19—N2—H2C	120.0
C9—N1—H1B	120.0	C19—N2—H2D	120.0
H1A—N1—H1B	120.0	H2C—N2—H2D	120.0
C2—C1—H1C	109.5	C12—C11—H11A	109.5
C2—C1—H1D	109.5	C12—C11—H11B	109.5
H1C—C1—H1D	109.5	H11A—C11—H11B	109.5
C2—C1—H1E	109.5	C12—C11—H11C	109.5
H1C—C1—H1E	109.5	H11A—C11—H11C	109.5
H1D—C1—H1E	109.5	H11B—C11—H11C	109.5
C1—C2—O1	113.0 (5)	O4—C12—C11	110.2 (4)
C1—C2—H2A	109.0	O4—C12—H12A	109.6
O1—C2—H2A	109.0	C11—C12—H12A	109.6
C1—C2—H2B	109.0	O4—C12—H12B	109.6
O1—C2—H2B	109.0	C11—C12—H12B	109.6
H2A—C2—H2B	107.8	H12A—C12—H12B	108.1
O2—C3—O1	121.8 (4)	O5—C13—O4	124.2 (4)
O2—C3—C4	126.7 (4)	O5—C13—C14	124.3 (3)
O1—C3—C4	110.9 (3)	O4—C13—C14	111.5 (3)
C3—C4—C5	114.2 (3)	C13—C14—C15	117.7 (3)
C3—C4—H4A	108.7	C13—C14—H14A	107.9
C5—C4—H4A	108.7	C15—C14—H14A	107.9
C3—C4—H4B	108.7	C13—C14—H14B	107.9
C5—C4—H4B	108.7	C15—C14—H14B	107.9
H4A—C4—H4B	107.6	H14A—C14—H14B	107.2
C10—C5—C6	119.1 (4)	C20—C15—C16	117.7 (3)
C10—C5—C4	122.0 (4)	C20—C15—C14	120.4 (3)
C6—C5—C4	118.8 (4)	C16—C15—C14	121.8 (4)
C7—C6—C5	118.8 (4)	C17—C16—C15	120.3 (4)
C7—C6—H6A	120.6	C17—C16—H16A	119.8
C5—C6—H6A	120.6	C15—C16—H16A	119.8
C8—C7—C6	122.6 (4)	C18—C17—C16	121.2 (4)
C8—C7—H7A	118.7	C18—C17—H17A	119.4
C6—C7—H7A	118.7	C16—C17—H17A	119.4
O3—C8—C7	126.1 (4)	O6—C18—C17	126.4 (3)
O3—C8—C9	114.9 (4)	O6—C18—C19	113.6 (3)
C7—C8—C9	119.0 (3)	C17—C18—C19	119.6 (3)
N1—C9—C10	126.8 (4)	C20—C19—C18	118.4 (3)
N1—C9—C8	115.3 (4)	C20—C19—N2	121.9 (3)
C10—C9—C8	117.7 (4)	C18—C19—N2	119.6 (3)
C5—C10—C9	122.7 (4)	C19—C20—C15	122.6 (3)
C5—C10—H10A	118.6	C19—C20—H20A	118.7
C9—C10—H10A	118.6	C15—C20—H20A	118.7
			
C3—O1—C2—C1	83.7 (6)	C13—O4—C12—C11	−171.1 (3)
C2—O1—C3—O2	12.7 (7)	C12—O4—C13—O5	1.6 (6)
C2—O1—C3—C4	−175.5 (4)	C12—O4—C13—C14	−176.5 (3)
O2—C3—C4—C5	−21.0 (7)	O5—C13—C14—C15	13.4 (6)
O1—C3—C4—C5	167.7 (4)	O4—C13—C14—C15	−168.5 (3)
C3—C4—C5—C10	−101.7 (5)	C13—C14—C15—C20	−102.0 (5)
C3—C4—C5—C6	76.3 (5)	C13—C14—C15—C16	74.0 (5)
C10—C5—C6—C7	1.0 (5)	C20—C15—C16—C17	−4.7 (6)
C4—C5—C6—C7	−177.0 (3)	C14—C15—C16—C17	179.2 (4)
C5—C6—C7—C8	−0.5 (6)	C15—C16—C17—C18	3.5 (6)
C6—C7—C8—O3	179.0 (3)	C16—C17—C18—O6	−173.4 (4)
C6—C7—C8—C9	−0.5 (5)	C16—C17—C18—C19	−1.5 (6)
O3—C8—C9—N1	−3.8 (4)	O6—C18—C19—C20	173.8 (3)
C7—C8—C9—N1	175.7 (3)	C17—C18—C19—C20	0.9 (5)
O3—C8—C9—C10	−178.7 (3)	O6—C18—C19—N2	−10.3 (5)
C7—C8—C9—C10	0.8 (5)	C17—C18—C19—N2	176.8 (3)
C6—C5—C10—C9	−0.6 (5)	C18—C19—C20—C15	−2.4 (5)
C4—C5—C10—C9	177.3 (3)	N2—C19—C20—C15	−178.2 (4)
N1—C9—C10—C5	−174.5 (4)	C16—C15—C20—C19	4.2 (6)
C8—C9—C10—C5	−0.3 (5)	C14—C15—C20—C19	−179.6 (3)

### Hydrogen-bond geometry (Å, °)

**Table:**

*D*—H···*A*	*D*—H	H···*A*	*D*···*A*	*D*—H···*A*
N1—H1B···O2^i^	0.86	2.37	3.097 (5)	143
N2—H2D···O5^ii^	0.86	2.42	3.222 (5)	155
O3—H3A···N2^iii^	0.82	2.23	2.978 (5)	152
O6—H6B···N1^iv^	0.82	2.31	3.015 (5)	145
C10—H10A···O6^ii^	0.93	2.49	3.414 (5)	174
N1—H1B···O3	0.86	2.12	2.517 (5)	108
N2—H2D···O6	0.86	2.33	2.641 (4)	102

Symmetry codes: (i) −*x*+2, −*y*+1, −*z*+1; (ii) −*x*+1, −*y*+2, −*z*+1; (iii) *x*+1, *y*−1, *z*; (iv) *x*, *y*+1, *z*.
